# End-of-Life Decisions: A Cross-National Study of Treatment Preference Discussions and Surrogate Decision-Maker Appointments

**DOI:** 10.1371/journal.pone.0057965

**Published:** 2013-03-05

**Authors:** Natalie Evans, H. Roeline Pasman, Tomás Vega Alonso, Lieve Van den Block, Guido Miccinesi, Viviane Van Casteren, Gé Donker, Stefano Bertolissi, Oscar Zurriaga, Luc Deliens, Bregje Onwuteaka-Philipsen, on behalf of EUROIMPACT

**Affiliations:** 1 Department of Public and Occupational Health, EMGO^+^ Institute, VU University Medical Center, Amsterdam, The Netherlands; 2 Public Health Directorate, Ministry of Health (Dirección General de Salud Pública, Consejería de Sanidad), Valladolid, Castille and León, Spain; 3 End-of-Life Care Research Group, Vrije Universiteit Brussel, Brussels, Belgium; 4 Clinical and Descriptive Epidemiology Unit, Cancer Prevention and Research Institute, (ISPO – L’Istituto per lo Studio e la Prevenzione Oncologica), Florence, Italy; 5 Scientific Institute of Public Health (WIV-ISP - Wetenschappelijk Instituut Volksgezondheid, Institut Scientifique de Santé Publique), Brussels, Belgium; 6 Netherlands Institute of Health Services Research (NIVEL - Nederlands instituut voor onderzoek van de gezondheidszorg), Utrecht, The Netherlands; 7 Italian Society of General Practice (SIMG – Società Italiana di Medicina Generale), Florence, Italy; 8 Public Health and Research General Directorate, Valencian Regional Health Administration, Valencia, Spain; 9 Higher Public Health Research Centre (CSISP - Centro Superior de Investigacion en Salud Pública), Valencia, Spain; 10 Spanish Consortium for Research on Epidemiology and Public Health (CIBERESP - El Consorcio de Investigación Biomédica de Epidemiología y Salud Pública), Madrid, Spain; Swiss Tropical & Public Health Institute, Switzerland

## Abstract

**Background:**

Making treatment decisions in anticipation of possible future incapacity is an important part of patient participation in end-of-life decision-making. This study estimates and compares the prevalence of GP-patient end-of-life treatment discussions and patients’ appointment of surrogate decision-makers in Italy, Spain, Belgium and the Netherlands and examines associated factors.

**Methods:**

A cross-sectional, retrospective survey was conducted with representative GP networks in four countries. GPs recorded the health and care characteristics in the last three months of life of 4,396 patients who died non-suddenly. Prevalences were estimated and logistic regressions were used to examine between country differences and country-specific associated patient and care factors.

**Results:**

GP-patient discussion of treatment preferences occurred for 10%, 7%, 25% and 47% of Italian, Spanish, Belgian and of Dutch patients respectively. Furthermore, 6%, 5%, 16% and 29% of Italian, Spanish, Belgian and Dutch patients had a surrogate decision-maker. Despite some country-specific differences, previous GP-patient discussion of primary diagnosis, more frequent GP contact, GP provision of palliative care, the importance of palliative care as a treatment aim and place of death were positively associated with preference discussions or surrogate appointments. A diagnosis of dementia was negatively associated with preference discussions and surrogate appointments.

**Conclusions:**

The study revealed a higher prevalence of treatment preference discussions and surrogate appointments in the two northern compared to the two southern European countries. Factors associated with preference discussions and surrogate appointments suggest that delaying diagnosis discussions impedes anticipatory planning, whereas early preference discussions, particularly for dementia patients, and the provision of palliative care encourage participation.

## Introduction

The nature of the patient-physician relationship has changed considerably over the last forty years with patient autonomy and participation in decision-making increasingly recognised [1]. For patients receiving end-of-life (EoL) care, participation includes preparation for possible future incapacity.

The most well known form of anticipatory decision-making is an advance directive. Advance directives are documents that outline treatments that a patient considers acceptable in the event that he or she can no longer communicate or that designate a surrogate decision-maker to make treatment choices on the patient’s behalf [2]. Research indicates that, even in the US, where advance directives are actively promoted and legally binding, uptake amongst the general public remains low, at around 20% [3], [4]. International studies and comparisons suggest even lower uptake [5], [6].

Advance directives however are just one aspect of anticipatory decision-making. The cornerstone of this is rather the process of patient-physician discussion regarding EoL treatment decisions [7]. Therefore, measures of patient-physician discussions about treatment preferences or the informal and formal appointment of surrogate decision-makers may be more appropriate indicators of patients’ involvement in treatment decisions than advance directive uptake [8], [9]. Moreover, due to cross-country differences in legal status and use of advance directives, such measures are particularly appropriate for international comparisons [5], [10].

Few studies have examined patient-physician EoL treatment preference discussions or patients’ appointment of surrogate decision-makers (written and verbal). Furthermore, results of these studies are difficult to compare due to differences in study population and question formulation. Previous studies have focused on the discussion of specific treatments [11]–[13], formal surrogate appointments (legal guardians or power of attorney) [14], [15] or on specific patient populations [11], [14]–[16].

This study examines GP-patient discussions of medical EoL treatment preferences and patients’ appointment of surrogate decision-makers in Italy, Spain, Belgium and the Netherlands. The study draws on information from representative GP sentinel networks about patient care in the last three months of life. In the four countries, almost all patients are registered with a GP [17] and GPs are instrumental in the delivery and coordination of EoL care [18]–[22].

Specific objectives include: to estimate and compare the prevalence of GP-patient medical EoL treatment preference discussions and patients’ appointment of surrogate decision-makers in four European countries; and to examine country specific factors associated with treatment preference discussions and surrogate appointments.

## Methods

### Study Design, Setting and Population

The study follows a cross-sectional, retrospective design. Participants from representative GP networks registered every patient death and described the patient and care characteristics using a standardised registration form.

In Spain, Belgium and the Netherlands, existing GP sentinel networks, used for epidemiological surveillance, took part in the study [18], [20], [23]. In Italy a network was created specifically for the study [24]. To avoid selecting GPs with a particular interest in EoL care, recruited GPs were not informed about the subject of the surveillance prior to participation [24].

In Belgium and the Netherlands the networks were nationwide and covered 1.75% and 0.8% of the population respectively. The Spanish network operated in two autonomous communities (Castile and León, and Valencia), covering 3.8% and 2.2% of the respective regional populations. The Italian network operated in nine local health districts and covered 4% of the population per health district. GPs registered deaths (aged 18 or older) from 01/01/2009 to 31/12/2010, apart from Spanish GPs who registered deaths from 01/01/2010 to 31/12/2010.

A total of 6,858 deaths were recorded. To include only patients who could have received EoL care, deaths registered as sudden and totally unexpected (n = 2243), or for which this information was missing (n = 97), were excluded. As the study concerns patient-GP discussions, only patients under their GP’s care were included (patients resident in their own or a family member’s home, or a care/residential home). Dutch nursing home patients, cared for by the nursing home physician, were excluded (n = 22). Patients whose main place of residence was ‘unknown’ (n = 28) or ‘other’ (often institutions outside the GP’s care) (n = 72) were also excluded. The final sample consisted of 4,396 deaths (Italy n = 1,808, Spain n = 379, Belgium n = 1,556, the Netherlands n = 653). Comparing the data with national data on non-sudden deaths (excluding Dutch nursing home deaths in the Netherlands) verified representativeness of all deaths (except for a slight underrepresentation of non-sudden hospital deaths and people under the age of 65 in Belgium, and women in the Netherlands) [24].

### Informed Consent, Patient Anonymity and Ethics Approval

After being informed of the objectives and procedures of the study, participating GPs gave written informed consent at the beginning of each registration year. Strict procedures regarding patient anonymity were employed during data collection and entry; every patient received an anonymous reference code from their GP and any identifying patient and GP data (such as date of birth, postcode and GP identification number) were replaced with aggregate categories or anonymous codes.

In Belgium the protocol of the study was approved by the Ethical Review Board of Brussels University Hospital of the Vrije Universiteit Brussel (2004). In Italy, ethics approval for data collection was obtained from the Local Ethical Committee ‘Comitato Etico della Azienda U.S.L. n. 9 di Grosseto’, Tuscany (2008). Ethical approval was not required for posthumous collection of anonymous patient data in the Netherlands [25], [26] or Spain [27]–[29].

### Measurement Instrument

The 2009/2010 EURO SENTI-MELC (European Sentinel Network Monitoring End-of-Life Care) form consisted of 21 structured questions about the patient’s demographic, health, and care characteristics in the last three months of life. Participants were requested to include information from hospital physicians and patient records.

Discussion of treatment preferences was determined from the two-part question, “Did the patient ever express specific wishes about a medical treatment that he/she would or would not want in the final phase of life?” And, if yes, “Did you ever speak to the patient about these wishes?” The current article focuses on the second part of this question. With regard to surrogate decision-maker appointments, the registration form included the item, “Did the patient ever express a wish about who was to make decisions regarding medical treatments or activities in his/her place in the event he/she would no longer be able to speak for him/herself?

The following independent variables, associated with anticipatory decision making in previous studies [20], [30], [31], were also collected: age; sex; cause of death; dementia diagnosis; residence in the last year of life; place of death; GP contacts; GP provision of palliative care (as defined by the GP); the importance of curative, life-prolonging and palliative care (on a 5-point Likert Scale); and whether the GP had discussed the primary diagnosis with the patient.

Most questions included in the registration form had been used in previous Dutch and Belgian studies [20], and had been subjected to extensive piloting [30], [33]. New questions were developed in collaboration with all partners. The final registration form underwent forward and backward translations from Dutch into English, from English into Italian and Spanish, and from Dutch into French and was piloted in each country (with 10 to 15 GPs) [24].

### Data Analysis

For each country, study population characteristics were assessed using descriptive statistics and differences between countries were assessed using Pearson’s chi-sq tests.

Prevalence of patient-GP treatment preference discussions and appointment of surrogate decision-makers were estimated per country using descriptive statistics. Differences between countries were examined using logistic regressions (controlling for study populations characteristics which differed significantly between countries).

Country specific factors associated with treatment preference discussions and surrogate appointments were examined through univariate and multivariable logistic regressions. Associations significant in univariate analyses were included in multivariable models. Stepwise backwards procedures were used (criteria for entry p<0.05 and for removal p>0.1) and residuals examined.

Continuous variables were transformed to be categorical (age, number of GP contacts). Cause of death was re-categorised as cancer or non-cancer. Furthermore, the treatment aims were dichotomised by combining “important” and “very important” in one category and other responses in another. All data analysis was carried out in SPSS version 18.

## Results

### Characteristics of the Study Population

Patient and care characteristics are shown in Table 1. The mean age of death was 80, 81, 79 and 77 for Italian, Spanish, Belgian and Dutch patients respectively. Although characteristics varied between countries, the most common cause of death was cancer (37–52%). Just under a third of patients in Italy, Spain and Belgium suffered from dementia (29–31%), compared with 13% of Dutch patients.

**Table 1:** Patients’ personal and care characteristics (n = 4,396)^a^.

		IT	ES	BE	NL	*p* value^b^
		n = 1808	n = 379	n = 1556	n = 653	
		n (%)	n (%)	n (%)	n (%)	
Age	< = 64	227 (13)	43 (11)	214 (14)	119 (18)	<0.001
	65–74	293 (16)	47 (12)	212 (14)	125 (19)	
	75–84	556 (31)	124 (33)	516 (33)	198 (30)	
	85>	732 (40)	165 (44)	602 (39)	211 (32)	
	Mean	79.6	80.5	79.0	77.0	
Sex	Male	844 (47)	202 (53)	712 (46)	304 (47)	0.075
	Female	964 (53)	177 (47)	840 (54)	342 (53)	
Cause of death	Cancer	820 (46)	147 (39)	581 (37)	339 (52)	<0.001
	Cardiovascular disease	371 (21)	63 (17)	226 (15)	101 (16)	
	Respiratory disease	129 (7)	53 (14)	168 (11)	50 (8)	
	Diseases of the nervous system	104 (6)	17 (5)	113 (7)	20 (3)	
	Stroke	177(10)	40 (11)	103 (7)	28 (4)	
	Other	163 (9)	56 (15)	363 (23)	112 (17)	
Patient diagnosed with dementia		520 (29)	112 (31)	478 (31)	84 (13)	<0.001
Place of death	Hospital	697 (39)	124 (33)	556 (36)	171 (28)	<0.001
	Residential or care home	163 (9)	46 (12)	479 (31)	112 (18)	
	Home (inclu. service flat) or with family	842 (47)	186 (50)	365 (24)	273 (44)	
	Palliative care unit/hospice	100 (6)	16 (4)	147 (10)	65 (10)	
	(Other n = 41)^c^					
Number of GP-patient contactsin the week before death	0	475 (26)	123 (32)	366 (24)	162 (25)	<0.001
	1 or 2	786 (43)	149 (39)	768 (49)	173 (26)	
	3>	547 (30)	107 (28)	422 (27)	318 (49)	
Number of GP-patient contactsin the second and third monthbefore death	0	145 (8)	73 (19)	127 (8)	130 (20)	<0.001
	1 or 2	972 (54)	222 (59)	1227 (79)	369 (57)	
	3>	691 (38)	84 (22)	202 (13)	154 (24)	
GP provided palliative care		995 (55)	232 (65)	787 (51)	374 (60)	<0.001
Treatment aim important orvery important	Curative treatment	322 (18)	91 (24)	468 (31)	141 (24)	<0.001
	Life prolongation	747 (42)	91 (24)	573 (39)	165 (28)	<0.001
	Palliative care	749 (42)	182 (48)	733 (51)	390 (65)	<0.001
GP and patient had discussedthe primary diagnosis		880 (49)	172 (50)	932 (60)	498 (78)	<0.001

a % of missing observations ranged from 0.3–4.5%.

b test of association: Pearson’s chi-sq.

c not included in statistical analyses - Patients for whom the main place of care in the last year of life was reported as “other” and Dutch patients in nursing homes were excluded from the analysis for reasons described in the methods section.

Approximately half of the Italian, Spanish and Dutch patients died at home (44–50%), compared with under a quarter of Belgian patients (24%). 24–32% of patients in the last week of life, and 8–20% of patients in the second and third months before death had no contact with their GP. GPs however provided palliative care to 51–65% of patients.

Curative treatment was important in the care of 18–31% of patients, prolonging life in 24–49% of cases and palliative care in 42–65% of cases. GPs had discussed the primary diagnosis with 49% of Italian, 50% of Spanish, 60% of Belgian and 78% of Dutch patients.

### Patient-GP Discussion of Medical EoL Treatment Preferences and Patient Appointment of a Surrogate Decision-maker

Table 2 shows the prevalence of treatment preference discussions and surrogate decision-maker appointments in the four countries.

**Table 2:** The prevalence of patient-GP communication about medical EoL treatment preferences and patient appointment of a surrogate decision-maker (n = 4,396)^a^.

		IT		ES		BE		NL	
		n = 1808		n = 379		n = 1556		n = 653	
		n (%)	Multivariable	n (%)	Multivariable	n (%)	Multivariable	n (%)	Multivariable
			OR (CI)^b^		OR (CI)^b^		OR (CI)^b^		OR (CI)^b^
Patient either discussed a treatment preference or appointed a surrogate		234 (13)	1	37 (10)	0.80 (0.54, 1.20)	487 (31)	**3.53 (2.84, 4.39)**	339 (52)	**6.02 (4.64, 7.81)**
Patient discussed a medical EoL treatment preference with their GP		173 (10)	1	26 (7)	0.83 (0.52, 1.33)	394 (25)	**3.80 (2.99, 4.83)**	304 (47)	**6.44 (4.88, 8.50)**
Patient appointed a surrogate decision-maker		110 (6)	1	20 (5)	0.93 (0.55, 1.57)	244 (16)	**2.78 (2.12, 3.64)**	187 (29)	**4.48 (3.32, 6.05)**
Manner of surrogate appointment	Only verbally	105 (6)	1	19 (5)	0.97 (0.57, 1.65)	191 (12)	**2.15 (1.62,2.86)**	128 (20)	**2.87 (2.08, 3.97)**
	In writing	4 (0)	c	1 (0)	c	53 (3)	c	53 (8)	c

a % of missing observations ranged from 0.3–1.2%.

b multivariable logistic regressions (forced enter). Dependent variables were ‘Patient did not discuss a medical EoL preference with GP or appoint a surrogate decision-maker’; ‘Patient discussed a medical EoL preference’; ‘Patient appointed a surrogate decision-maker’; ‘Patient appointed a surrogate decision-maker in writing’; and ‘Patient only appointed a surrogate decision-maker verbally’. Independent variables included country (OR and p-value shown), age, cause of death, dementia diagnosis; place of death; the number of contacts with the GP in the last week and in the second and third months before death; GP palliative care provision; the importance of curative, life-prolonging and palliative care as treatment aims and if the GP had discussed the primary diagnosis. The results of the multivariate logistic regressions were compared with equivalent univariate analyses (not shown) to check for any major differences in the magnitude or direction of associations.

c Too few patients in this category to conduct a logistic regression.

A minority of patients from all countries (10–31%), except the Netherlands (52%), had either discussed treatment preferences or appointed a surrogate decision-maker. GP-patient discussion of treatment preferences had taken place with 10% of Italian, 7% of Spanish, 25% of Belgian and 47% of Dutch patients. Furthermore, 6% of Italian, 5% of Spanish, 16% of Belgian and 29% of Dutch patients had appointed (either verbally or in writing) a surrogate decision-maker.

Multivariable logistic regressions revealed a strong association between country and both treatment preference discussions and surrogate appointments. The odds of discussing treatment preferences with a GP were over six times higher for a Dutch patient, and almost four times higher for a Belgian patient, compared with an Italian patient. Similarly, the odds of appointing a surrogate decision-maker were over four times higher for a Dutch patient, and almost three times higher for a Belgian patient, than for an Italian patient. There were no significant differences in the odds of GP-patient discussion of treatment preferences or appointment of surrogate decision-makers between Italy and Spain. Surrogate appointment was entirely verbal in Italy and Spain and most frequently verbal in the Netherlands and Belgium.

### Factors Associated with Discussion of a Medical EoL Treatment Preference

Table 3 shows the factors associated with GP-patient discussion of EoL treatment preferences in univariate and multivariable analyses. The multivariable models revealed country specific associations. Diagnosis of dementia was negatively associated with treatment preference discussions in Belgium and the Netherlands. Palliative care unit (PCU) deaths were positively associated with preference discussions compared with hospital deaths in Belgium. Furthermore, in Belgium, more frequent GP contact in the last week of life was positively associated with preference discussions, and in both Belgium and the Netherlands more frequent contact in the second and third months before death was positively associated with preference discussions. GP provision of palliative care was positively associated with preference discussions in all countries and the recognition of palliative care as an important/very important treatment aim was positively associated with preference discussions in Belgium. Previous GP-patient discussion of the primary diagnosis was positively associated with preference discussions in all countries.

**Table 3:** Characteristics associated with a patient having discussed a medical EoL treatment preference with their physician in univariate and multivariable analyses (n = 4,396)^a^.

*Preference discussed*	IT (n = 1808)		ES (n = 379 )		BE (n = 1556)		NL (n = 653)	
	Logistic regression^b^		Logistic regression^b^		Logistic regression^b^		Logistic regression^b^	
	Univariate	Multivariable	Univariate	Multivariable	Univariate	Multivariable	Univariate	Multivariable
	OR (95% CI)	OR (95% CI)	OR (95% CI)	OR (95% CI)	OR (95% CI)	OR (95% CI)	OR (95% CI)	OR (95% CI)
***Age***								
< = 64	**2.37 (1.48, 3.79)**	d	**4.32 (1.19, 15.71)**	d	**1.71 (1.22, 2.42)**	d	1.14 (0.72, 1.79)	c
65–74	**2.27 (1.46, 3.52)**		**4.68 (1.36, 16.11)**		**1.44 (1.01, 2.04)**		1.34 (0.86, 2.09)	
75–84	1.38 (0.92, 2.08)		2.81 (0.93, 8.43)		1.06 (0.80, 1.40)		1.03 (0.70, 1.53)	
85>	**1**		1		**1**		1	
***Sex***								
Male	1.24 (0.90, 1.69)	c	1.03 (0.46, 2.29)	c	1.10 (0.88, 1.39)	c	0.73 (0.54, 1.00)	c
***Cause of death***								
Respiratory disease	**1**	d	1	c	**1**	d	**1**	d
Cardiovascular disease	1.05 (0.50, 2.21)		0.83 (0.16, 4.31)		**2.04 (1.23, 3.41)**		1.31 (0.64, 2.68)	
Cancer	1.71 (0.87, 3.37)		1.91 (0.53, 6.88)		**3.14 (2.00, 4.93)**		**2.42 (1.29, 4.53)**	
Diseases of the nervous system	0.73 (0.26, 2.08)		1.04 (0.10, 10.73)		0.97 (0.50, 1.88)		0.98 (0.33, 2.93)	
Stroke	0.56 (0.22, 1.47)		0.43 (0.04, 4.27)		0.79 (0.39, 1.62)		0.32 (0.09, 1.07)	
Other	1.12 (0.48, 2.61)		0.94 (0.18, 4.89)		1.19 (0.73, 1.96)		1.14 (0.56, 2.32)	
***No dementia diagnosis***	**3.64 (2.23, 5.93)**	d	**3.62 (1.06, 12.31)**	d	**4.18 (3.03, 5.78)**	**2.30 (1.52, 3.50)**	**3.04 (1.80, 5.13)**	**2.28 (1.18, 4.44)**
***Place of death***								
Hospital	1	c	1	c	**1**	**1**	**1**	d
Residential or care home	0.82 (0.44, 1.52)		e		0.84 (0.61, 1.15)	0.96 (0.62, 1.49)	**3.32 (1.98, 5.56)**	
Home (inclu. service flat) or with family	1.03 (0.73, 1.45)		2.69 (0.98, 7.39)		**3.09 (2.30, 4.16)**	**1.71 (1.15, 2.53)**	**5.83 (3.77, 9.00)**	
Palliative care unit/hospice	0.93 (0.45, 1.93)		1.57 (0.17, 14.39)		**2.39 (1.61, 3.56)**	**1.93 (1.22, 3.04)**	**2.42 (1.32, 4.44)**	
***Number of GP-patient contacts***								
Last week before death								
0	**1**	1	1	d	**1**	**1**	**1**	1
1 or 2	0.86 (0.55, 1.33)	0.80 (0.50, 1.26)	2.16 (0.66, 7.05)		**1.67 (1.20, 2.32)**	**1.71 (1.14, 2.55)**	**2.28 (1.38, 3.77)**	0.93 (0.47, 1.86)
3>	**2.12 (1.41, 3.19)**	1.53 (0.97, 2.39)	**3.76 (1.17, 12.03)**		**3.37 (2.38, 4.76)**	**2.93 (1.81, 4.75)**	**8.47 (5.36, 13.39)**	1.88 (0.94, 3.75)
Second and third months before death								
0	**1**	d	**1**	d	**1**	**1**	**1**	**1**
1 or 2	1.79 (0.81, 3.96)		0.47 (0.16, 1.37)		**1.89 (1.13, 3.17)**	**1.78 (0.90, 3.51)**	**2.25 (1.46, 3.47)**	**2.09 (1.13, 3.84)**
3>	**2.77 (1.25, 6.11)**		1.71 (0.60, 4.87)		**4.10 (2.31, 7.27)**	**2.36 (1.13, 4.95)**	**3.38 (2.06, 5.56)**	**2.04 (1.04, 3.99)**
***GP provided palliative care***	**2.59 (1.81, 3.69)**	**2.07 (1.40, 3.07)**	**4.53 (1.33, 15.41)**	**4.96 (1.43, 17.25)**	**2.27 (1.79, 2.88)**	**1.54 (1.11, 2.14)**	**8.81 (6.00, 12.93)**	**4.40 (2.57, 7.54)**
***Care aim important or very important***								
Curative treatment	0.84 (0.55, 1.30)	c	1.18 (0.48, 2.89)	c	**0.64 (0.49, 0.83)**	d	0.84 (0.57, 1.23)	c
Life prolongation	0.80 (0.58, 1.10)	c	0.75 (0.27, 2.04)	c	0.88 (0.69, 1.12)	c	0.88 (0.61, 1.25)	c
Palliative care	**1.65 (1.20, 2.26)**	d	2.17 (0.94, 4.99)	c	**1.68 (1.32, 2.14)**	**1.45 (1.09, 1.93)**	**2.42 (1.71, 3.43)**	d
***Primary diagnosis discussed***	**8.40 (5.31, 13.28)**	**7.47 (4.71, 11.87)**	**29.45 (3.94, 220.01)**	**28.59 (3.81, 214.63)**	**7.22 (5.22, 10.00)**	**4.66 (3.13, 6.94)**	**11.15 (6.33, 19.63)**	**5.66 (2.51, 12.75)**

Values for which p<0.05 are highlighted in **bold.**

a 0.3–4.5% of values for each characteristic were not provided by the GP (missing values).

b Backwards stepwise logistic regression - dependent variable ‘Patient discussed a medical EoL preference with their GP’.

c Not entered into logistic regression.

d Removed during logistic regression.

e No patients in the category had discussed a medical EoL preference with their GP (odds ratio of 0).

### Factors Associated with Patient Appointment of a Surrogate Decision-maker

The factors associated with surrogate decision-maker appointments in univariate and multivariable analyses are presented in Table 4. Country specific associations were revealed in the multivariable models. Surrogate appointments were negatively associated with male patients in the Netherlands. In Spain PCU/hospice deaths were positively associated with surrogate appointments compared with hospital deaths.

**Table 4:** Characteristics associated with patients' appointment of a surrogate decision-maker by country in iunivariate and multivariable analyses (n = 4,396)^a^.

*Surrogate appointed*	IT (n = 1808)		ES (n = 379 )		BE (n = 1556)		NL (n = 653)	
	Logistic regression^b^		Logistic regression^b^		Logistic regression^b^		Logistic regression^b^	
	Univariate	Multivariable	Univariate	Multivariable	Univariate	Multivariable	Univariate	Multivariable
	OR (95% CI)	OR (95% CI)	OR (95% CI)	OR (95% CI)	OR (95% CI)	OR (95% CI)	OR (95% CI)	OR (95% CI)
***Age***								
< = 64	1.20 (0.64, 2.25)	c	1.25 (0.24, 6.44)	c	1.12 (0.74, 1.68)	c	1.13 (0.69, 1.85)	c
65–74	1.55 (0.91, 2.65)		3.13 (0.91, 10.77)		0.73 (0.46, 1.16)		1.11 (0.68, 1.80)	
75–84	1.22 (0.76, 1.96)		1.56 (0.51, 4.77)		0.86 (0.62, 1.19)		0.97 (0.63, 1.50)	
85>	1		1		1		1	
***Sex***								
Male	1.25 (0.85, 1.83)	c	1.30 (0.52, 3.26)	c	0.96 (0.73, 1.26)	c	**0.54 (0.38, 0.77)**	**0.43 (0.28, 0.65)**
***Cause of death***								
Respiratory disease	1	c	1	c	**1**	d	1	c
Cardiovascular disease	0.93 (0.35, 2.42)		1.37 (0.22, 8.51)		1.22 (0.71, 2.11)		1.19 (0.54, 2.62)	
Cancer	1.65 (0.70, 3.89)		1.89 (0.40, 8.92)		1.33 (0.83, 2.13)		1.61 (0.81, 3.21)	
Diseases of the nervous system	0.82 (0.23, 3.00)		e		0.87 (0.44, 1.73)		0.88 (0.24, 3.19)	
Stroke	1.34 (0.48, 3.74)		e		0.48 (0.21, 1.11)		0.56 (0.16, 1.95)	
Other	1.46 (0.53, 4.06)		2.55 (0.47, 13.76)		0.83 (0.49, 1.40)		0.89 (0.40, 1.97)	
***No dementia diagnosis***	1.60 (1.00, 2.56)	c	1.32 (0.47, 3.73)	c	1.30 (0.96, 1.77)	c	**1.85 (1.04, 3.29)**	d
***Place of death***								
Hospital	**1**	d	**1**	**1**	**1**	d	**1**	d
Residential or care home	0.51 (0.20, 1.30)		e	e	1.19 (0.83, 1.70)		**2.20 (1.23, 3.94)**	
Home (inclu. service flat) or with family	1.11 (0.73, 1.69)		1.52 (0.51, 4.48)	2.20 (0.67, 7.19)	**1.76 (1.23, 2.52)**		**3.34 (2.07, 5.40)**	
Palliative care unit/hospice	1.41 (0.64, 3.11)		**9.44 (2.18, 40.84)**	**12.49 (2.54, 61.49)**	**1.96 (1.23, 3.12)**		**1.99 (1.00, 3.93)**	
***Number of GP-patient contacts***								
Last week before death								
0	**1**	d	1	c	**1**	**1**	**1**	**1**
1 or 2	1.03 (0.62, 1.73)		1.21 (0.42, 3.49)		**1.90 (1.26, 2.87)**	**1.86 (1.20, 2.89)**	1.22 (0.64, 2.36)	0.57 (0.26, 1.23)
3>	**1.69 (1.01, 2.82)**		0.91 (0.27, 3.06)		**2.99 (1.95, 4.59)**	**3.14 (1.99, 4.95)**	**6.43 (3.75, 11.02)**	**3.27 (1.74, 6.15)**
Second and third months before death								
0	1	c	1	c	**1**	d	**1**	d
1 or 2	1.59 (0.63, 4.05)		1.06 (0.28, 3.96)		1.72 (0.93, 3.17)		**2.77 (1.58, 4.84)**	
3>	2.23 (0.88, 5.70)		2.00 (0.50, 8.05)		**2.83 (1.43, 5.58)**		**4.28 (2.34, 7.84)**	
***GP provided palliative care***	**1.53 (1.02, 2.28)**	d	2.11 (0.69, 6.50)	c	**1.66 (1.25, 2.19)**	d	**3.93 (2.60, 5.95)**	d
***Care aim important or very important***								
Curative treatment	0.89 (0.53, 1.49)	c	1.03 (0.36, 2.91)	c	0.87 (0.64, 1.18)	c	0.75 (0.49, 1.15)	c
Life prolongation	1.07 (0.72, 1.58)	c	1.71 (0.66, 4.43)	c	1.09 (0.82, 1.45)	c	0.72 (0.48, 1.08)	c
Palliative care	**1.78 (1.21, 2.63)**	**1.67 (1.12, 2.48)**	1.60 (0.64, 4.00)	c	**1.54 (1.15, 2.04)**	**1.49 (1.11, 2.01)**	**2.38 (1.60, 3.55)**	**1.62 (1.02, 2.57)**
***Primary diagnosis discussed***	**2.15 (1.43, 3.23)**	**1.92 (1.27, 2.89)**	**5.74 (1.64, 20.09)**	**5.68 (1.58, 20.37)**	**2.69 (1.95, 3.71)**	**2.56 (1.83, 3.58)**	**5.98 (3.14, 11.38)**	**5.37 (2.31, 12.49)**

Values for which p<0.05 are highlighted in bold.

a 0.3–4.5% of values for each characteristic were not provided by the GP (missing values).

b Backwards stepwise logistic regression - dependent variable ‘Patient had appointed a surrogate decision-maker’.

c Not entered into logistic regression.

d Removed during logistic regression.

e No patients in the category had appointed a surrogate decision-maker (odds ratio of 0).

More frequent patient-GP contact in the last week before death was positively associated with surrogate appointments for Belgium and the Netherlands. Furthermore, the importance of palliative care was positively associated with surrogate appointments in Belgium, the Netherlands and Spain.

Previous discussion of the primary diagnosis between the patient and the GP was positively associated with surrogate appointments in all four countries.

## Discussion

These data reveal that a minority of patients from all countries, with the exception of the Netherlands, had either discussed treatment preferences or appointed a surrogate decision-maker. Furthermore, there are important cross-country differences in prevalence of discussions and surrogate appointments, which were highest in the Netherlands, followed by Belgium, with no significant differences between Spain and Italy.

The single most important patient or care factor associated with treatment preferences discussions in all countries, and with surrogate appointments in the Netherlands and Italy, was prior GP-patient discussion of the primary diagnosis.

### Differences between Countries

There are notable differences between northern and southern European countries with a lower prevalence of treatment preference discussions and surrogate appointments in Italy and Spain. Considering the strong association between discussion of the primary diagnosis and both preference discussions and surrogate appointments, these cross-country differences are linked to lower levels of primary diagnosis discussion in Italy and Spain compared with the Netherlands and Belgium. Previous studies have also reported limited disclosure [9], [31], [34], [35] and discussion of EoL treatment preferences [11], [36] in the two southern European countries.

Meñaca et al [9], in a review of EoL care and culture in Italy, Spain and Portugal, highlighted the influence of Catholicism on disclosure of diagnoses and prognoses. Catholic teaching permits the gradual disclosure of “truth” to terminal patients in a way that does not destroy hope [37]. Meñaca [9] also found that although advance directives have a strong legal status in Spain (in contrast to Italy) in practice physicians are more guided by the principle of beneficence [9]. In Italy, it has been suggested that physicians’ concern about distress caused by EoL treatment discussions leads them to delay or avoid such discussions [37].

Belgium, in contrast, although nominally Catholic, has more in common with the Netherlands. The process of legalization of euthanasia in both countries engendered open public debate on EoL issues [5], [38]. A higher frequency of anticipatory decision-making in the Netherlands and Belgium may therefore be expected considering the importance of self-determination and the open discussion of death and dying. This is especially true in the Netherlands, where patients prioritize autonomy and control in the dying process [39], [40]. Cross-country studies have repeatedly found that Dutch physicians’ more frequently discuss EoL issues than their European counterparts [13], [41], [42].

Family members’ opposition to full-disclosure of primary diagnosis, the so called “conspiracy of silence”, has also been said to contribute to low levels of disclosure in both Italy and Spain [9]. A lack of disclosure and subsequent EoL discussions may also impact patients’ appointment of surrogate decision-makers. Equally, such appointments may be deemed unnecessary if family members are considered *de facto* proxies. This may contribute to the lower prevalence of surrogate appointments compared with preference discussions in all countries, particularly in Spain and Italy, which are often seen as more family orientated.

An additional consideration concerns patients’ wishes for information. A systematic review of EoL communication reported that studies from northern European countries report higher levels of desired information amongst patients than studies from the south of Europe [43]. Although the desire for diagnosis and prognosis information may not be as common amongst patients and the general public in Italy and Spain compared to northern European countries; in general, the proportion reported to prefer full disclosure is still greater than the proportion that receives full disclosure in clinical practice [9], [44].

A GP’s responsibility for EoL care also varies between the four countries. In the Netherlands there is a strong focus on GP EoL care provision: GPs are primarily responsible for generalist EoL care provision and have easy access to palliative care guidelines and consultation [45], [46]. In Belgium, Spain and Italy however provision is more often shared with palliative care home teams [21], [47], [48]. Furthermore GPs have a ‘gatekeeper’ role (coordinating all referrals to specialist services) in the Netherlands and Spain, but not in Belgium and Italy.

A further explanation for the strong cross-country differences lies in the amount of palliative care training physicians receive. A survey of physicians from Belgium, Denmark, Italy, the Netherlands, Sweden and Switzerland revealed that the percentage of physicians who had undertaken formal palliative care training was lowest in Italy and highest in the Netherlands [49]. Palliative care training may improve EoL communication skills and may contain specific EoL communication training.

### Country Specific Factors Associated with Treatment Preference Discussions and Surrogate Appointments

A number of patient and care characteristics were associated with treatment preferences discussions. As mentioned previously GP-patient discussion of the primary diagnosis was strongly associated with both treatment preferences and surrogate appointments. In addition, dementia diagnosis was associated with less frequent discussion of treatment preferences in Belgium and the Netherlands. Timely discussions are a priority for patients with dementia. A related issue is the early diagnosis of dementia. Research shows that 50–66% of patients with dementia are not diagnosed with the condition by primary care physicians [50]. GPs are recommended to begin preference discussions as soon as mental capacity decline is detected.

Frequency of contact with GPs, GP provision of palliative care and the importance of palliative care as a treatment aim were also associated with preference discussions and surrogate appointments.

Palliative care unit and home deaths were associated with treatment preference discussions in Belgium. This may reflect an emphasis on palliative home care in Belgium [5] and discussion of preferences in the palliative care sector. In Spain, surrogate appointment was associated with PCU and hospice deaths. Indeed, for Spain, ‘place of death’ was the factor most strongly associated with surrogate appointments in the multivariable model; suggesting that, for Spanish patients, surrogate appointment is specifically related to specialist inpatient palliative care.

Interestingly, in the Netherlands patient surrogate appointment was more frequent amongst female patients. This may indicate a greater reluctance amongst GPs to discuss surrogate appointments with male patients or of male patients to assign decision-making responsibilities. Men are also more likely to have a living partner, so may feel less need to appoint a surrogate decision-maker [51]. Why this should only be significant in the Netherlands and not the other countries is, however, unclear.

### Strengths and Limitations

This is the first population-based study to estimate the prevalence of medical EoL treatment discussions and patients’ appointment of surrogates in the Netherlands, Belgium, Italy and Spain. The use of the same study design amongst representative GP sentinel networks in each country provided robust and comparable data. Bias was avoided by selecting GPs with no specific interest in EoL care. As most people in each country are registered with a GP, representative samples of non-sudden deaths were obtained. A strength of the retrospective design is that a representative sample of the palliative care population could be identified.

The study was, however, subject to a number of limitations. Although GPs completed registration forms on a weekly basis, there may have been some recall bias. In addition, GPs may have provided socially desirable answers especially concerning items that reflect on their own care competencies; particularly high levels of GP provision of palliative care for example were reported in all four countries. Furthermore, the study reports the discussion of treatment preferences according to the GP. Patients and physicians may differ in their perception of what constitutes the “discussion” of treatment preferences and patients may have discussed preferences with other health professionals.

The Spanish and Italian sentinel networks were not nationwide, although they were representative of the areas they covered (Italy was representative for the largest statistical regions). Dutch nursing home residents were excluded from analyses and there was a slight underrepresentation of non-sudden hospital deaths and people under the age of 65 in Belgium and a slight underrepresentation of females in the Netherlands. Some sudden deaths in hospitals may also have been missed by GPs in Spain and Italy. However, due to a lack of national data on place of death, this could not be tested. The survey also relied on GPs to report care in other settings, although GPs were asked to maximize information from other sources. In addition, GPs’ characteristics were unavailable; preventing examination of associations with GP characteristics.

Finally, the study only examines the prevalence of treatment preference discussions and surrogate appointments and some associated factors. Further qualitative research on the patient-physician communication process may help in understanding the complex reasons for between country differences.

### Conclusions

Discussion of both medical EoL treatment preferences and surrogate appointments were highest in the Netherlands, followed by Belgium, with no significant differences between Spain and Italy. A number of factors related to the discussion of the primary diagnosis, patient’s mental capacity and specialist or generalist palliative care were associated with treatment discussions and surrogate appointments.

These findings suggest that the process of planning for the EoL often starts with the discussion of the primary diagnosis: if avoided or delayed, opportunities for patient participation in decision-making may be missed. Communication training for physicians can help change attitudes towards diagnosis disclosure [52], [53]. Ideally training would also highlight the right of a patient not to receive such information if he or she so wishes; such a preference however must be stated by the patient and not assumed *a priori* by the physician.

Furthermore, early preference discussions for all patients, particularly those with dementia or cognitive decline, and the provision of palliative care support patients’ participation in EoL decision-making.
